# A diagnosis model of dementia *via* machine learning

**DOI:** 10.3389/fnagi.2022.984894

**Published:** 2022-09-07

**Authors:** Ming Zhao, Jie Li, Liuqing Xiang, Zu-hai Zhang, Sheng-Lung Peng

**Affiliations:** ^1^School of Computer Science, Yangtze University, Jingzhou, China; ^2^Department of Ophthalmology, The First Affiliated Hospital of Yangtze University, Jingzhou, China; ^3^Department of Creative Technologies and Product Design, National Taipei University of Business, Taipei, Taiwan

**Keywords:** dementia, machine learning, bagging, principal component analysis, diagnosis model

## Abstract

As the aging population poses serious challenges to families and societies, the issue of dementia has also received increasing attention. Dementia detection often requires a series of complex tests and lengthy questionnaires, which are time-consuming. In order to solve this problem, this article aims at the diagnosis method of questionnaire survey, hoping to establish a diagnosis model to help doctors make a diagnosis through machine learning method, and use feature selection method to select important questions to reduce the number of questions in the questionnaire, so as to reduce medical and time costs. In this article, Clinical Dementia Rating (CDR) is used as the data source, and various methods are used for modeling and feature selection, so as to combine similar attributes in the data set, reduce the categories, and finally use the confusion matrix to judge the effect. The experimental results show that the model established by the bagging method has the best effect, and the accuracy rate can reach 80% of the true diagnosis rate; in terms of feature selection, the principal component analysis (PCA) has the best effect compared with other methods.

## Introduction

In recent years, China has slowly entered a deeply aging society, with the elderly accounting for 14% of the total population. By 2033, China will enter a super-aging society with 22% of the elderly population. Then around 2060, the proportion of aging will reach 35%, which means that by 2060, 1 in 3 Chinese will be over 65 years old. In an aging society, the health care of the elderly has become an important issue, in which dementia clearly occupies a very important position. Alzheimer’s disease is the most common type of dementia, accounting for approximately 70% of all dementias (GBD 2019 Dementia Forecasting Collaborators, 2022). For dementia, the earlier it is detected, the earlier treatment can be initiated. But as the population continues to age and the number of people with dementia continues to increase, dementia screening has become an urgent problem.

Since the beginning of the 20th century, machine learning has made remarkable achievements in various fields. The combination of machine learning and the medical field is particularly remarkable. Especially in the severe epidemic period, the use of machine learning methods can help doctors to quickly identify lung CT images and make a diagnosis (Elaziz et al., 2020; Prakash et al., 2020; Afshar et al., 2021; Aboghazalah et al., 2022; Das et al., 2022; Elkamouny and Ghantous, 2022; Shiri et al., 2022; Sun et al., 2022). There are also many studies and applications of machine learning in dementia, researchers also summarize many applications of machine learning and deep learning in dementia (Ahmed et al., 2018; Miah et al., 2021). Alashwal et al. (2019) found patterns in patients that were difficult for medical practitioners to spot by using clustering methods in unsupervised learning in machine learning, and identified several features of the transition from early to late stages of dementia. Alexiou et al. (2017) used a Bayesian model to predict early dementia, and used the model to correlate and evaluate biomarkers to output predicted probabilities. Alickovic and Subasi (2019) used histograms to convert brain images into feature vectors, and passed these features into classifiers constructed by machine learning methods such as random forests to achieve automatic detection of Alzheimer’s disease. An et al. (2020) used an ensemble learning method to build a dementia classification model, and the obtained model was better than any single algorithm. Ansari et al. (2019) used a deep learning network to analyze the EEG (Electroencephalogram) features of the incoming network. At the end of the network, a random forest classifier was used to classify the output, and the final detection accuracy could reach 77%. Bloch and Friedrich (2019) used volumetric features from multiple magnetic resonance imaging (MRI) scans to classify Alzheimer’s disease, and the resulting best model had a test classification accuracy of 75.49%.

The above-mentioned methods basically analyze pathological images, which require the elderly to go to the hospital for some professional examinations to obtain relevant data, which is time-consuming and labor-intensive. And in recent years, affected by the epidemic, people usually do not go to the hospital before they have obvious symptoms. Therefore, this article aims to use a questionnaire to conduct a preliminary examination of dementia, and use machine learning methods to establish a dementia diagnosis model. Prior to this, Trambaiolli et al. (2011); Williams et al. (2013), Broman et al. (2022), and Khan et al. (2022) have used machine learning combined with some questionnaires to detect and classify dementia. Based on these studies, this article optimizes some questionnaire questions by cooperating with clinicians, using a variety of machines. Learning methods to model and extract features, so as to combine similar attributes in the data set, reduce the number of questions in the questionnaire, and achieve rapid screening of dementia.

The rest of this article is arranged as follows:

Section “Related work” introduces dementia and related knowledge of machine learning that will be used in this article. In section “Experiment and analysis,” experiment and analysis of experimental results will be explained. In section “Conclusion and future work,” summary and prospects of the research will be given.

## Related work

In this section, we will explain the theory and terminology used in the article, including an explanation of dementia-related terms, an introduction to machine learning, and the algorithms used in this research.

### Dementia

The most common type of dementia is Alzheimer’s disease in the elderly. The typical initial symptom is memory impairment. The patient forgets what has just happened (poor short-term memory), while memory from older times (long-term memory) is relatively unaffected in the early stages of the disease. Dementia affects language skills, comprehension, motor skills, short-term memory, ability to identify everyday objects, reaction time, personality, executive ability, and problem-solving skills. Even if there are no signs of mental decline, delusions are common (15–56% of Alzheimer’s types), such as doubting that the person in the mirror is someone else.

Symptoms of dementia also include changes in personality or behavior. Many patients with a final diagnosis of dementia had intense confusional symptoms early in their hospitalization. Older adults may also have symptoms of mental changes due to other medications, surgery, infections, lack of sleep, an abnormal diet, dehydration, changing places, or a personal crisis. Because most patients with dementia may have symptoms of insanity. Although the symptoms of confusion may be alleviated by close care, improvement of living environment and diet; Psychiatric drugs can also help stabilize mood, reduce hallucinations and delusions, or control impulse. But at present, drugs have not been able to slow down brain degeneration. Dementia patients are often accompanied by depression, and it is best to be diagnosed and treated by professionals.

### Mild cognitive impairment

The definition of mild cognitive impairment (MCI) is as follows:

(1) Subjective memory impairment.
(2) Objective memory impairment.
(3) Poor memory compared with people of the same age and education level.
(4) Normal cognition and daily life function.
(5) Not dementia, has not reached the degree of dementia.

### Clinical dementia rating

The Clinical Dementia Rating (CDR) is mainly aimed at patients with Alzheimer’s disease. By asking their caregivers, an overall assessment of daily living and cognitive function is carried out to define the severity of the disorder. CDR contains six projects: Memory (M), Orientation (O), judgment-problem-solving (J), Community affairs (C), Home hobbies (H), and Personal care (P), with five severity levels from 0 to 3: 0 stands for normal Health, 0.5 for suspected or Mild impairment, 1 for Mild dementia, 2 for Moderate dementia, 3 represents Severe dementia. The evaluator observed the patient’s current performance, and based upon the information provided by the caregiver, took memory as the main item score, and sense of orientation, judgment and problem-solving, community affairs, home and hobbies and personal care as the secondary items scores, and then calculated the CDR score according to the rules. Among them, the Normal category, although it represents normal in this article, the patients who had gone to the hospital for treatment more or less have problems with memory, so the Normal here does not mean that the patients really have no problems, but it is analyzed in this scale is normal. The CDR used in this article is a revised version of the collaborating doctors after years of clinical experience, and the questions are shown in Table 1.

**TABLE 1:** Questions in the clinical dementia rating.

Question number	Question content	Options				
M01	Are cognitive functions (e.g., memory, thinking, and judgment) significantly worse than before?	Yes	No			
M02	Do you forget the name of your spouse or children?	Yes	No			
M03	Has cognitive decline affected daily life, social interaction and work?	Yes	No			
M04	Do the symptoms of cognitive function fluctuate greatly, or even get worse within a day?	Yes	No			
M05	Do you often lose things?	Yes	Never or occasionally			
M06	Do you often forget what you said recently?	Yes	Never or occasionally			
M07	Do you find it difficult to learn how to use tools and equipment?	Yes	No			
M08	Do you often forget what happened recently?	Yes	Never or occasionally			
M09	Do you ask the same questions or say the same things over and over again?	Yes	No			
M10	Will you cherish the past (often mention the past)?	Yes	No			
M12	Do you forget familiar things (such as place of origin, address, and occupation)?	Yes	No			
O02	Will you forget the correct year and month?	Yes	No			
O03	Is it difficult to remember when to date?	Yes	No			
O05	Do you get lost in familiar surroundings, such as near your home?	Yes	No			
O06	Can’t figure out where you are?	Yes	No			
O07	Do you often mistake your son for your husband and your daughter for your sister?	Yes	Never or occasionally			
J01	Do you often behave inappropriately when dealing with advance and retreat (such as attending weddings and funerals of friends and relatives)?	Yes	No			
J02	Does judgment often have difficulty? (e.g., falling into a trap, a scam, and buying inappropriate gifts)	Yes	Never or occasionally			
J03	Is it difficult to handle complex finances (banking, paying bills, writing checks)?	Yes	No			
J04	Do you feel that your work ability or professional skills have deteriorated?	Yes	No			
J05	Will it be difficult to deal with big and small affairs inside and outside the home?	Yes	No			
J06	Is it obviously more difficult to operate daily necessities than before? (e.g., using a telephone, a remote control, or a microwave oven, etc.)	Yes	No			
C01	Go shopping (go out shopping to buy gifts and vegetables, etc.)	Complete by oneself	Every time you go shopping, you need someone to accompany you or you won’t buy it at all.			
C02	Money handling capacity	Normal	Can handle routine purchases, but needs help dealing with banks or cannot handle money			
C03	The ability to use the phone, such as making or receiving calls	Normal	Can only answer the phone, but can’t dial the phone or completely need help			
C04	Cooking food (or preparing a table of dishes such as cooking, ordering or cooking)	Normal	Need someone else to cook, set or order the meal			
C05	Household maintenance (simple housework such as housekeeping, cleaning)	Normal	All household chores need help from others			
C06	Laundry (or handling personal correspondence such as mailing and receiving)	Normal	Completely dependent on others			
C07	Outings (ride or ride, drive to destination)	Normal	Need assistance or escort			
C08	Self-medication	Normal	Self-administration or not self-administration if the amount of medication to be taken is prepared in advance			
C09	Difficulty in the above activities, the patient is due to physical or mobility impairment	Yes	No			
H01	Are you still engaged in routine activities? (For example, walking, chanting Buddha, going to temples, worshipping, praying, and going to church, etc.) or common hobbies or interests? (For example, dancing, playing cards, mahjong, karaoke, mallet, etc., ball, playing with grandchildren, etc.)	As usual.	A little less	A lot less	Almost no	Not at all, in my room all day
P01	Eating	At a reasonable time, you can eat your immediate food with chopsticks		Need someone to help put on and take off eating aids or only eat with a spoon		Inability to self-feed or take too long
P02	Transfer between wheelchair and bed	Can be completed independently	Need a little help or verbal instruction	Able to sit up from bed on their own, but still needs help when moving	You can only sit up when others help you
P03	Personal hygiene	Able to wash face, wash hands, brush teeth and comb hair independently	Need help from others
P04	To the restroom	Can go to the toilet and be self-assembled, and will not stain clothes	Need to help keep balance, tidy clothes or use toilet paper	Need help from others
P05	Bath	Can be done independently	Need help from others
P06	Walk up and down stairs	Can be done independently	Need a little help or verbal guidance	Unable to go up and down stairs
P07	Put on and take off clothes	Clothes, shoes and accessories that can be put on and taken off by oneself	With the help of others, you can complete more than half of the movements by yourself	Need help from others
P08	Walk more than 50 m on the flat ground	Can walk independently	Need a little support or guidance	Can’t walk, but can operate the wheelchair independently	Need help from others
P09	Stool control	No incontinence and self-administration of suppositories	Occasional incontinence or needing help using suppositories	Need to be handled by others
P10	Urinary control	No urinary incontinence day or night	Occasional incontinence or need help	Need to be handled by others

### Very early dementia screening scale (AD-8)

The Early Dementia Screening Scale (AD-8) is a simple tool for screening dementia. It was invented by Washington University and put forward in 2005. It can screen out very mild dementia symptoms and is widely used in the world. The scale contains eight questions. The long-term caregiver observes the individual’s long-term changes and fills in the answer, or the individual can fill in the answer by themselves. The scoring method is to fill in “yes, there is a change” and get 1 point, and fill in “no, there is no change” and get 0 point. If the long-term caregiver cannot assess the individual condition, fill in “I don’t know,” then this question will not be scored. If the total score is greater than or equal to 2 points, the subject needs to go to the hospital for further evaluation. The problems of the AD-8 scale are shown in Table 2.

**TABLE 2:** The problems of the AD-8 scale.

Question	Yes, there is a change (1 point)	No, no change (0 points)	Don’t know (no credit)
1. Difficulty in judgment: e.g., falling into a trap or scam, making a bad financial decision, and buying a gift that is inappropriate for the recipient			
2. Decreased interest in activities and hobbies			
3. Repeat the same questions, stories, and statements			
4. Difficulty learning how to use tools, equipment, and gadgets. For example: TV, stereo, remote control, air conditioner, washing machine, water heater, microwave oven, etc.			
5. Forget the correct month and year			
6. Difficulty dealing with complex finances. For example: personal or family balance of payments, bills of payment, income tax, etc.			
7. Difficulty remembering appointment times			
8. Has persistent thinking and memory problems			
Total			

### Machine learning

Machine learning is a multi-field interdisciplinary subject involving probability theory, statistics, approximation theory, convex analysis, algorithm complexity theory and other disciplines. It specializes in how computers simulate or realize human learning behaviors to acquire new knowledge or skills, and to reorganize existing knowledge structures to continuously improve their performance. In the current era of big data, machine learning is mainly used to find rules from data and build models, and then use the models to predict unknown data. When the input data is larger, the model continuously adjusts to make more accurate predictions.

The machine learning methods used in this article include Bagging and C4.5 decision tree. The C4.5 decision tree is an extension and optimization of the ID3 algorithm, which introduces improvements such as information gain rate. The algorithm mentioned above will be briefly explained below.

#### ID3

ID3 is a decision tree algorithm whose structure is based on information theory proposed by Shannon. In information theory, entropy represents the expected value of a random variable, and in the ID3 algorithm, it is a pointer that determines the importance of the variable. The following is an introduction to the entropy algorithm in ID3:

Calculation of data volume before test


(1) $$\text{info}\,\left(T\right)=-\sum_{i=1}^{m}\frac{\mathrm{freq}\,\left(C_{i},T\right)}{\left|T\right|}\,{\text{log}}_{2}\,\left(\frac{\mathrm{freq}\,\left(C_{i},T\right)}{\left|T\right|}\right)$$

*T*: A collection.

|*T*| : The amount of data in the set *T*.

*C_i*: Categories in the set,*i*=1,2,…,*m* (*m*: number of categories)

*freq*(*C*_*i*_,*T*): The number of categories of data in the set *T*.

Calculation of data volume after test


(2) $${\text{info}}_{X}\,\left(T\right)=\sum_{i=1}^{p}\frac{\left|T_{i}\right|}{\left|T\right|}\,\text{info}\,\left(T_{i}\right)$$

*T_i* : Subset of *T* set after testing against variable *X*, *i*=1,2,…,*p*,*X* ∈ {*X*_1_,*X*_2_,…,*X*_*p*_}

The algorithm of ID3 is developed based on the concept of information theory. The decision of nodes is determined by information gain, and the concept is that the amount of data before the test is subtracted from the amount of data after the test.


(3) $$\text{Gain}\,\left(X\right)=\text{info}\,\left(T\right)-{\text{info}}_{X}\,\left(T\right)$$

#### C4.5 decision tree

The C4.5 algorithm is a classic algorithm for generating decision trees, and it is an extension and optimization of the ID3 algorithm. The C4.5 algorithm has improved the ID3 algorithm. The main improvements are as follows:

(1) Using the information gain rate to select the partition features overcomes the deficiency of the information gain selection, but the information gain rate has a preference for the attributes with a small number of possible values.
(2) Ability to handle discrete and continuous attribute types, that is, to discretize continuous attributes.
(3) Ability to handle training data with missing attribute values.
(4) Pruning in the process of constructing the tree.

And the information gain ratio is calculated as follows:


(4) $$\mathrm{Gain}\,\mathrm{Ratio}\,\left(X\right)=\frac{\mathrm{Gain}\,\left(X\right)}{\mathrm{Splitinfo}\,\left(X\right)}$$

Among them


(5) $$\text{Splitinfo}\,\left(X\right)=\sum_{i=1}^{m}\frac{\left|T_{i}\right|}{\left|T\right|}\,\log_{2}\left(\frac{\left|T_{i}\right|}{\left|T\right|}\right)$$

#### Bagging

**FIGURE 1:**
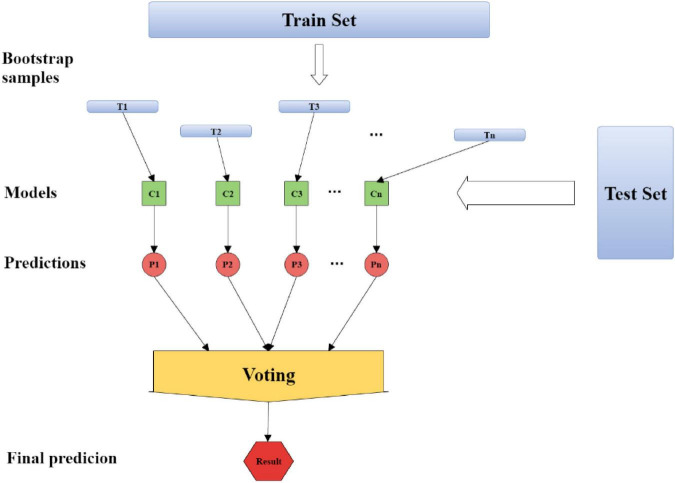
The process of Bagging algorithm.

Bagging (Bootstrap Aggregating) is a kind of ensemble learning. The ensemble algorithm is a method of combining multiple weak classifiers into strong classifiers in a certain combination. First, 60% of the data set is used as the training set, and 40% of the data set is used as the test set. About 60% of the data is randomly and repeatedly extracted from the training set to establish *T_n* sets of training data, and the *T_n* sets of training data are used. Build *C_n* models from the training data, substitute the data from the test set into *C_n* groups of models to get *P_n* answers, and finally get the results by voting or averaging for the *n* answers. The process of Bagging algorithm is shown in Figure 1.

### Principal components analysis

One of the main directions of research in this article is to reduce the number of questions in the questionnaire, which can be understood as reducing the number of features. This article will use three methods to measure the importance of each problem, namely information gain, information gain ratio, and PCA. Among them, the information gain and the information gain ratio have been introduced in the previous introduction of C4.5. Therefore, in this part, we will introduce PCA.

Principal component analysis is a very effective way to reduce dimensions. When analyzing data, it is often necessary to deal with a number of interrelated variables, transform interrelated variables into independent linear combinations, and explain the whole data structure with a few variables.

Assume that the original data *X* consists of the following:


(6) $$\text{X}=\begin{array}{c} \begin{array}{ccc} X_{1} & X_{2} & \ldots \end{array}\,\begin{array}{cc} X_{P} & \end{array}\\ {\left[\begin{array}{ccc} x_{11} & x_{12} & \ldots \,x_{1\,p}\\ x_{21} & x_{22} & \ldots \,x_{2\,p}\\ \begin{array}{c} x_{31}\\ \begin{array}{c} \vdots \\ x_{n\,1} \end{array} \end{array} & \begin{array}{c} x_{32}\\ \begin{array}{c} \vdots \\ x_{n\,2} \end{array} \end{array} & \begin{array}{c} \ldots \,x_{3\,p}\\ \begin{array}{c} \vdots \\ x_{n\,p} \end{array} \end{array} \end{array}\right]}_{n\times p} \end{array}$$

*p* is the number of variables, *n* is the number of samples, *X*_1_,*X*_2_,…,*X*_*P*_ are variables; The calculation process of the main components is divided into the following steps

(1) Data standardization


(7) Xi*=(Xi-Xi¯)σXi,i=1,2,….,p-1,p

Where Xi* is the standardized data, $\bar{X_{i}}=\frac{1}{n}\,\sum_{j=1}^{n}x_{i\,j}$ is the average of *X*_*i*_, $\sigma_{X_{i}}=\sqrt{\frac{1}{n}\,\sum_{j=1}^{n}{\left(x_{i\,j}-\bar{X_{i}}\right)}^{2}}$ is the standard deviation of *X_i_*.

(2) Calculate the correlation coefficient matrix between variables.


(8) $$R=\left(\begin{array}{ccc} r_{11} & r_{12} & \begin{array}{cc} \ldots & r_{1\,p} \end{array}\\ \begin{array}{c} r_{21}\\ \vdots \end{array} & \begin{array}{c} r_{22}\\ \vdots \end{array} & \begin{array}{c} \begin{array}{cc} \ldots & r_{2\,p} \end{array}\\ \begin{array}{cc} \ldots & \vdots \end{array} \end{array}\\ r_{p\,1} & r_{p\,2} & \begin{array}{cc} & r_{p\,p} \end{array} \end{array}\right)$$

(9) $$r_{i\,j}=\frac{{\left(X_{i}^{*}\right)}^{T}\,X_{j}^{*}}{\left(n-1\right)},i,j=1,2,\ldots ,p$$

*R* is the correlation coefficient matrix, and *r*_*ij*_ is the correlation coefficient.

(3) Calculate eigenvalues and eigenvectors

Substitute the correlation coefficient matrix into the characteristic equation, and solve the *p* eigenvalues _λ1,λ2,λ3_,…,λ_*P*_, and _λ1_ > λ_λ2_ > _λ3_ > … > λ_*P*_≥0.


(10) det⁢(R-λ⁢I)=0

where *R* is the correlation coefficient matrix, λ is the eigenvalue, and I is the identity matrix. Use _λ1,λ2,λ3_,…,λ_*p*_ to calculate the corresponding eigenvectors *V*_1_,*V*_2_,*V*_3_,…,*V*_*p*_.

(4) Select the number of principal component variables

Observe the cumulative ratio. In the experimental process, if the cumulative ratio is above 0.8, the effect is very good.

## Experiment and analysis

### Data set and experimental environment

The software used in this article is weka, version 3.8.1, and its full name is waikato environment for knowledge analysis. This software is a machine learning software written in Java, which integrates a large number of algorithms and has the characteristics of simple operation and powerful functions.

The data set is the diagnostic information collected from the hospital, which includes the patient’s gender, age, education level, problems, and diagnosis results. Here we call it Data A for short. Table 3 shows the date, total number, gender, age, and education level of Data A. The date is from 07/09/2015 to 14/04/2017. There are 1,565 people, and the number of female patients is larger than that of male patients. There were only 293 patients below the age of 65, and only 422 between the ages of 65 and 75. However, the number of people above the age of 75 increased sharply to 845. It can be found that the number of people at primary school level was the largest, reaching 1,178. The higher the level of education, the lower the number of patients. Data A contains 42 questions and diagnosis results. There are five categories of diagnosis results. Table 4 shows the number of people in each category. The answers to 33 questions are 0, 1, and the answers to 6 questions are 0, 1, 2, and 2. The answers to 1 question are 0, 1, 2, 3, and the answers to 1 question are 0, 1, 2, 3, 5. Table 5 illustrates the mean and standard deviation of the 42 questions in Data A.

**TABLE 3:** Attributes of data set A.

Date	07/09/2015–14/04/2017	
Total people		1,565
Sex	Male	656
	Female	909
Age	23–103	
	Age ≤ 65	293
	65 < Age ≤ 75	422
	Age 76	845
Education level	0–19 years	
	Primary school level (1–6 years)	1178
	Secondary school level (7–12 years)	288
	Advanced level (12–20 years)	99

**TABLE 4:** Diagnostic results.

State	Number of people
Normal	83
Uncertain dementia	397
Mild dementia	347
Moderate dementia	493
Severe dementia	246

**TABLE 5:** Average and standard deviation of the problem.

Question	Options	Mean	Standard deviation	Question	Options	Mean	Standard deviation
M01	0, 1	0.9016	0.29795	C01	0, 1	0.47476	0.49952
M02	0, 1	0.26965	0.44392	C02	0, 1	0.55783	0.4968
M03	0, 1	0.56805	0.49551	C03	0, 1	0.45176	0.49783
M04	0, 1	0.16294	0.36943	C04	0, 1	0.4901	0.50006
M05	0, 1	0.70032	0.45826	C05	0, 1	0.4377	0.49626
M06	0, 1	0.70671	0.45542	C06	0, 1	0.46454	0.4989
M07	0, 1	0.75655	0.4293	C07	0, 1	0.54633	0.49801
M08	0, 1	0.64153	0.4797	C08	0, 1	0.58275	0.49326
M09	0, 1	0.6492	0.47737	C09	0, 1	0.05304	0.22418
M10	0, 1	0.43387	0.49577	H01	0, 1, 2, 3, 5	1.65367	1.61955
M12	0, 1	0.26518	0.44157	P01	0, 1, 2	1.51757	0.73494
O02	0, 1	0.60511	0.48898	P02	0, 1, 2, 3	2.10032	1.25089
O03	0, 1	0.59617	0.49082	P03	0, 1	0.72077	0.44877
O05	0, 1	0.39233	0.48843	P04	0, 1, 2	1.36422	0.86284
O06	0, 1	0.32204	0.46741	P05	0, 1	0.59105	0.4918
O07	0, 1	0.1623	0.36884	P06	0, 1, 2	1.22812	0.91714
J01	0, 1	0.15911	0.36589	P07	0, 1, 2	1.37444	0.83121
J02	0, 1	0.57444	0.49459	P08	0, 1, 2, 3	2.09265	1.24585
J03	0, 1	0.56358	0.4961	P09	0, 1, 2	1.41406	0.837
J04	0, 1	0.56805	0.49551	P10	0, 1, 2	1.3623	0.83696
J05	0, 1	0.46837	0.49916				
J06	0, 1	0.4262	0.49468				

**FIGURE 2:**
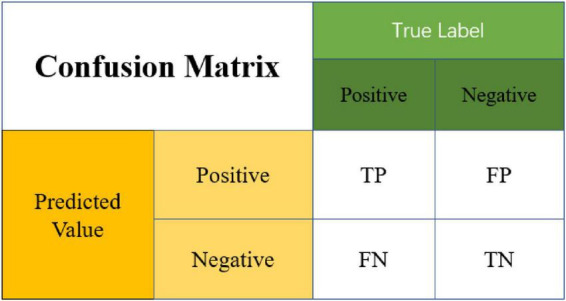
Confusion matrix diagram.

In order to verify the accuracy of the method, this article uses a confusion matrix to test the accuracy of the model (see Figure 2). It can judge whether the predicted value matches the actual value. The characteristic is that the classified category can be clearly seen.

The meaning of each item in the figure is explained as follows:

True-Positive (TP): The predicted value is Positive, and the actual value is also judged to be Positive.

False-Negative (FN): The predicted value is Negative, but the actual value is judged to be Positive.

False-Positive (FP): The predicted value is Positive, but the actual value is judged to be Negative.

True-Negative (TN): The predicted value is Negative, and the actual value is also judged as Negative.

Through this figure, its performance can be tested according to the following indicators, and it is hoped that the test algorithm can obtain the highest accuracy and true positive rate (TPR):


(11) $$A\,c\,c\,u\,r\,a\,c\,y=\frac{T\,P+T\,N}{\text{TP}+\text{TN}+\text{FN}+\text{FP}},T\,P\,R=\frac{T\,P}{\text{TP}+\text{FN}}$$

### Algorithm comparison

**FIGURE 3:**
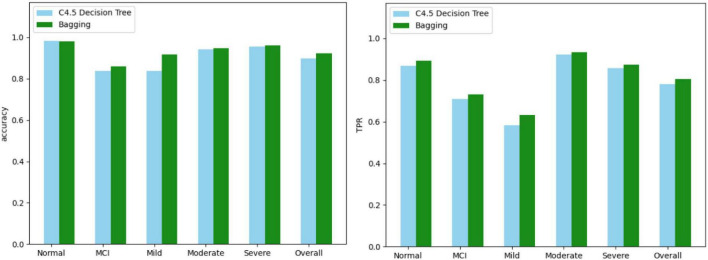
Comparison results of two algorithms.

In addition to the questions and results, Data A also includes gender, age, and education level. Here we only take all 42 different questions and results for analysis. The results are not calculated according to the CDR formula, but are re-diagnosed by physicians referring to the CDR questionnaire, and there are five result categories. In this part we substitute Data Set B into both algorithms and use the confusion matrix to see the effect. We combine the categories with similar attributes, reduce the categories, and use the confusion matrix to check the effect again. We choose C4.5 decision tree and Bagging to calculate, and use confusion matrix to calculate the accuracy and TPR of each category. Figure 3 shows the comparison results of the two algorithms.

### Decision scale analysis

#### Comparison of three algorithms

**FIGURE 4:**
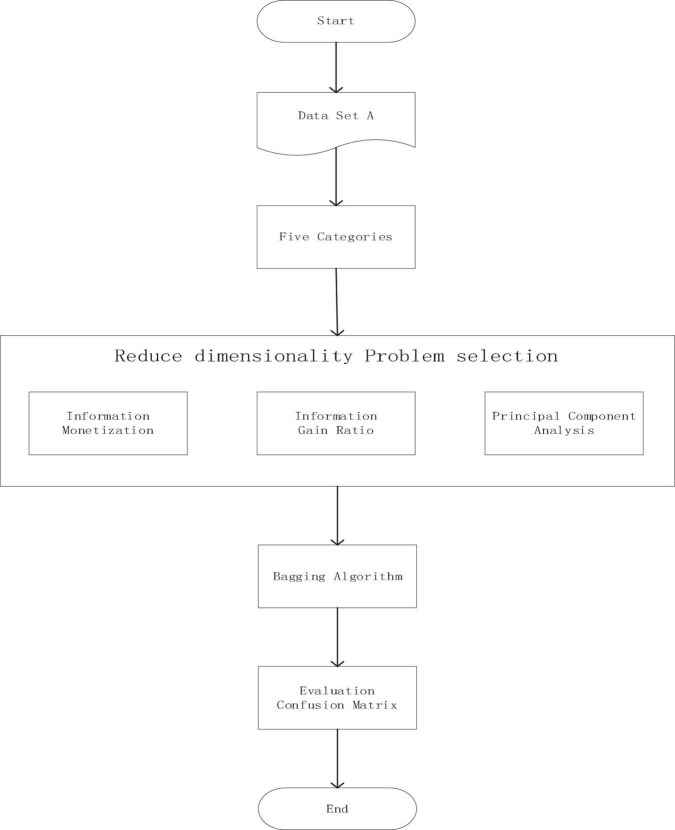
Flow chart of principal component extraction experiment.

In this part, we choose three algorithms (information gain, gain ratio, and PCA) to get the importance of 42 questions, and then delete the questions based on them, and watch the effect with the confusion matrix. Then, we choose the best algorithm and compare the eight questions selected with the eight questions in AD-8. The flow chart is shown in Figure 4.

**FIGURE 5:**
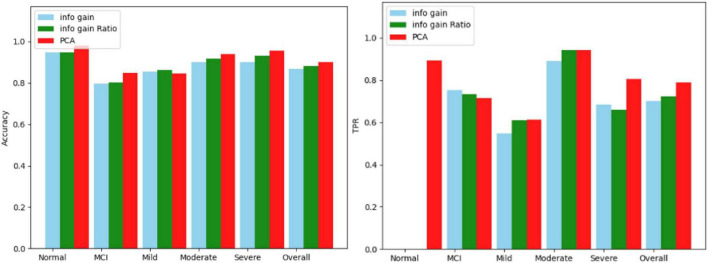
Comparison results for 19 questions.

**FIGURE 6:**
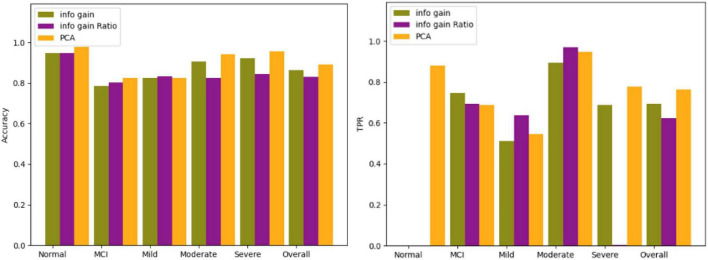
Comparison results for 14 questions.

**FIGURE 7:**
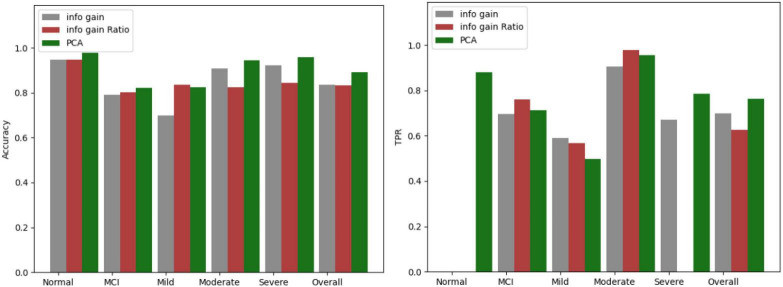
Comparison results for 12 questions.

**FIGURE 8:**
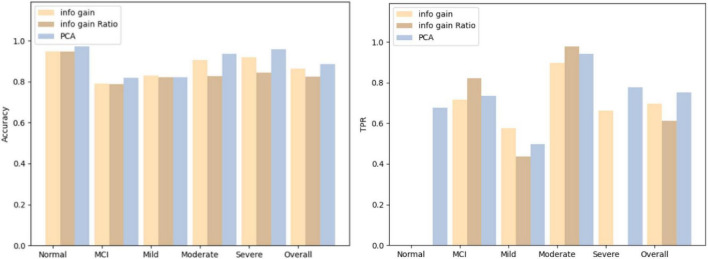
Comparison results for 9 questions.

The three screening methods individually pick out the questions with the highest scores, substitute the questions into the Bagging algorithm, and then use the confusion matrix to test the accuracy of each category. According to the number of variables in PCA, the number of questions is selected and compared four times, which is 19 questions, 14 questions, 12 questions, and 9 questions. The comparison results of the three algorithms are shown in Figures 5–8.

#### Comparison with AD-8

**FIGURE 9:**
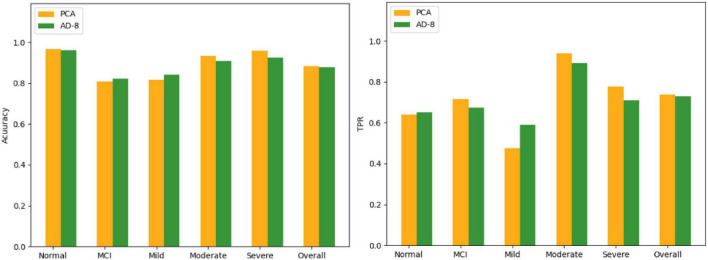
Comparison of PCA and AD-8 results.

In the CDR there are eight questions that are very similar to the AD-8, and in the AD-8 the answer is “yes, changed” or “no, no change,” while in the CDR the answer is quite different. Pick out the problems similar to AD-8 from the CDR, and then use the eight problems screened out by PCA to input them into the Bagging algorithm, and then use the confusion matrix for comparison. The eight problems selected are shown in Table 6. See Figure 9 for a comparison of the results, showing that both have the same degree of accuracy.

**TABLE 6:** Eight questions screened by PCA.

Algorithm	Question number							
PCA	M01	M05	M09	O07	J01	J02	C06	H01
AD-8	M01	M09	O02	O03	J02	J03	J06	H01

## Conclusion and future work

In the experimental part, it can be seen that the effect of the Bagging algorithm is the best, and the accuracy is above 80%. In the Normal category, although the C4.5 decision tree has a higher accuracy than Bagging, in TPR, Bagging is better than the decision tree, and TPR is what we value more. In the selection of important features, the effect of PCA is better than that of information profit or information profit ratio, especially in the Normal category, the TPR of information profit or information profit ratio is 0, both of which will be all Normal misidentification is unacceptable to us. And compared with AD-8, the effect of PCA in the five categories is also better.

Although medical databases are widely used now, the data on dementia is very scarce. It is hoped that in the future, a database will be established to collect a large amount of dementia data. The more data, the better the model there will be, to help patients understand their own situation and seek medical treatment earlier. In addition, we also hope to expand the fields for collecting data in medical databases. The more data there are, the more extensive the research topics will be, and the personal data of patients should be removed. And these data is used only for research purposes. In addition, more kinds of algorithms can be used in the future, be it fuzzy logic or neural network, or even deep learning algorithms. There will be a combination of categories and a way to deal with imbalanced data, and there should be different results.

## Data availability statement

The raw data supporting the conclusions of this article will be made available by the authors, without undue reservation.

## Author contributions

Z-HZ: resources. LX: supervision. MZ: funding acquisition. JL: writing—original draft preparation. MZ and S-LP: writing—review and editing. All authors have read and agreed to the published version of the manuscript.
